# A Phytochemical Constituent, (E)-Methyl-Cinnamate Isolated from *Alpinia katsumadai* Hayata Suppresses Cell Survival, Migration, and Differentiation in Pre-Osteoblasts

**DOI:** 10.3390/ijms21103700

**Published:** 2020-05-24

**Authors:** Kyung-Ran Park, Hanna Lee, MyoungLae Cho, Hyung-Mun Yun

**Affiliations:** 1 Department of Oral and Maxillofacial Pathology, School of Dentistry, Kyung Hee University, Seoul 02453, Korea; rudfks282@naver.com; 2 National Development Institute of Korean Medicine, Gyeongsan 38540, Korea; iiihanna@nikom.or.kr (H.L.); meanglae@nikom.or.kr (M.C.)

**Keywords:** phytomedicine, *Alpinia katsumadai* Hayata, (E)-methyl-cinnamate, osteoblast, apoptosis, MAPKs

## Abstract

Background: (E)-methyl-cinnamate (EMC), a phytochemical constituent isolated from *Alpinia katsumadai* Hayata, is a natural flavor compound with anti-inflammatory properties, which is widely used in the food and commodity industry. However, the pharmacological effects of methyl-cinnamate on pre-osteoblasts remain unknown. This study aimed to investigate the pharmacological effects and mechanisms of EMC in pre-osteoblast MC3T3-E1 cells (pre-osteoblasts). Methods: Cell viability and apoptosis were evaluated using the MTT assay and TUNEL staining. Cell migration and osteoblast differentiation were examined using migration assays, as well as alkaline phosphatase activity and staining assays. Western blot analysis was used to examine intracellular signaling pathways and apoptotic proteins. Results: EMC decreased cell viability with morphological changes and increased apoptosis in pre-osteoblasts. EMC also induced the cleavage of Poly (ADP-ribose) polymerase (PARP) and caspase-3 and reduced the expression of anti-apoptotic proteins. In addition, EMC increased TUNEL-positive cells in pre-osteoblasts, decreased the activation of mitogen-activated protein kinases, and suppressed cell migration rate in pre-osteoblasts. Subsequently, EMC inhibited the osteoblast differentiation of pre-osteoblasts, as assessed by alkaline phosphatase staining and activity assays. Conclusion: These findings demonstrate that EMC has a pharmacological and biological role in cell survival, migration, and osteoblast differentiation. It suggests that EMC might be a potential phytomedicine for treating abnormalities of osteoblast function in bone diseases.

## 1. Introduction

*Alpinia katsumadai* Hayata is widely used in traditional Chinese medicine to treat emesis and gastric disorders [1]. The seeds of *A. katsumadai* have been used as an antioxidant and a stomachic [2]. It has been reported that they possess various bioactive compounds, including flavonoids, stilbenes, chalcones, monoterpenes, and sesquiterpenoids [3]. (E)-methyl-cinnamate (EMC), isolated from the seeds of *A. katsumadai*, is a methyl ester synthesized by cinnamic acid methyltransferase from cinnamic acid. Cinnamic acid is an antioxidant phytochemical agent and is known to be a safe flavoring used in the food industry [4,5]. The following pharmacological properties of EMC have been demonstrated: antibacterial and anti-fungal [6,7]. This includes being antispasmodic and myorelaxant for anti-neuropathic pain and anti-inflammatory effects [8,9]. EMC was also shown to inhibit adipocyte differentiation in 3T3-L1 cells [10]. However, there is no information on the pharmacological effects and biological actions of EMC in osteoblasts.

Osteoblasts are specialized cells derived from mesenchymal stem cells (MSCs) [11]. MSCs differentiate into osteoblasts via the Smad1/5/8 and Runt-related transcription factor 2 (RUNX2) pathways by osteogenic factors, such as BMP. Some osteoblasts are surrounded by the mineralized bone matrix and differentiate into osteocytes [11,12]. Apoptotic processes play critical roles in osteoblasts to maintain the mineralized and dynamic bone tissues in bone physiology and disease [13,14,15]. In addition, the migration and differentiation of osteoblasts into the region of bone formation are important for the synthesis and secretion of bone proteins, and the mineralization of bone tissues [16,17,18]. Therefore, the dysregulation of osteoblast function is etiologically important in several bone diseases, such as osteoporosis, osteopetrosis, and Paget’s disease [14,19,20,21].

In the present study, we investigated the pharmacological and biological effects of EMC on apoptosis, migration, and differentiation in pre-osteoblast MC3T3-E1 cells (pre-osteoblasts).

## 2. Results

### 2.1. EMC Decreases Cell Survival and Induces Morphological Changes in Pre-Osteoblasts

**Figure 1:**
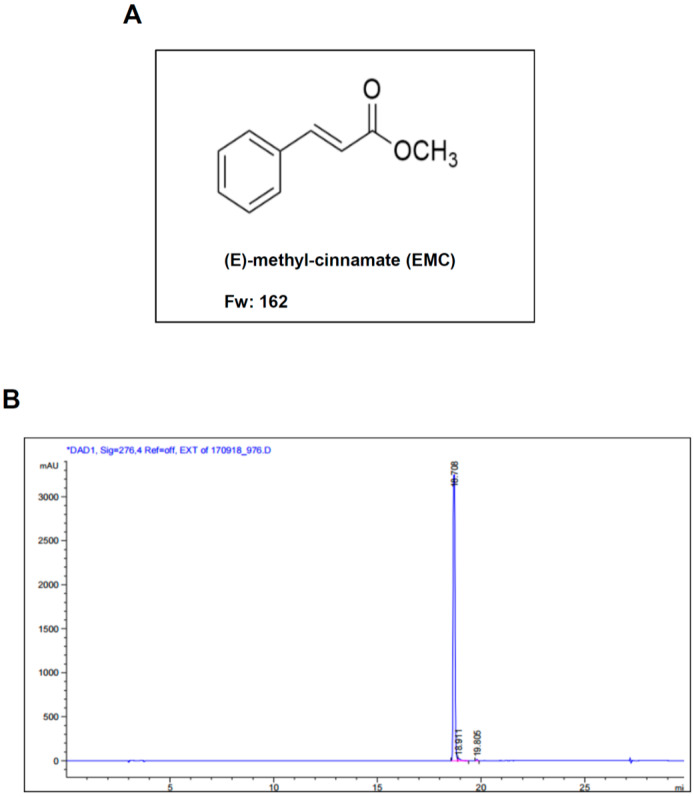
Chemical structure and High-performance liquid chromatography (HPLC) chromatogram of EMC. (**A**) Chemical structure of (E)-methyl-cinnamate (EMC) isolated from the seeds of *Alpinia katsumadai* Hayata. (**B**) HPLC chromatogram of EMC. The results are representative of three independent experiments.

**Figure 2:**
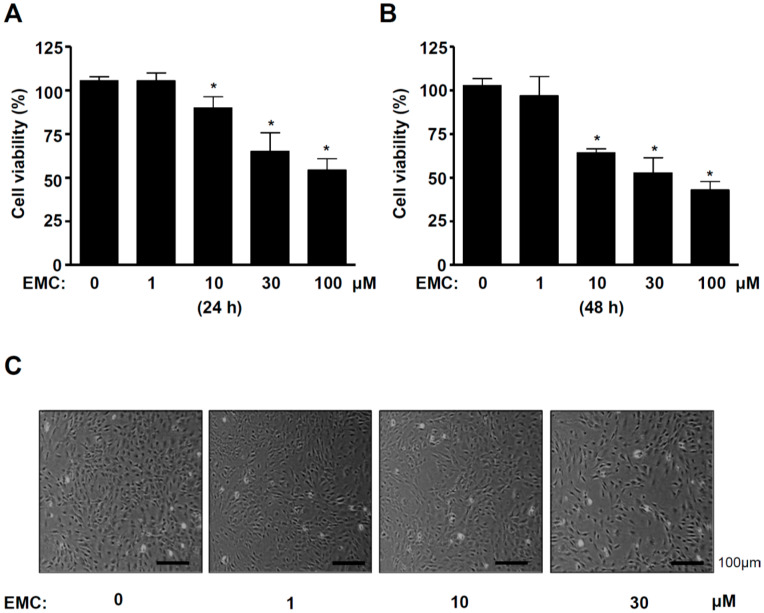
EMC decreases cell survival and induces morphological changes in pre-osteoblasts. (**A**,**B**) Pre-osteoblasts were treated with EMC (1, 10, 30, and 100 µM) for 24 h (**A**) and 48 h (**B**). Then, cell viability was measured by the MTT assay. (**C**) The morphological changes were observed under the phase contrast microscopy after treatment with EMC for 48 h. The data are expressed as the mean ± S.E.M. of experiments. * (*p* < 0.05) indicates statistically significant differences compared to the control.

(E)-methyl-cinnamate (EMC) was isolated from the seeds of *A. katsumadai* using HPLC to evaluate its effect on pre-osteoblasts. The structure and HPLC chromatogram of EMC are shown in Figure 1A,B. Pre-osteoblasts were initially treated with various doses of EMC for 24 and 48 h. Cell proliferation was measured using the 3-[4,5-dimethylthiazol-2-yl]-2,5-diphenyltetrazolium bromide (MTT) assay. EMC significantly inhibited cellular proliferation in a time- and dose-dependent manner (Figure 2A,B). EMC also induced a morphologic change into small and protruding cell shapes with shrinkage in a dose-dependent manner (Figure 2C). Based on these results, moderate concentrations of EMC (10 and 30 µM) were selected to be used in all subsequent experiments.

### 2.2. EMC Induces Apoptosis in Pre-Osteoblasts

**Figure 3:**
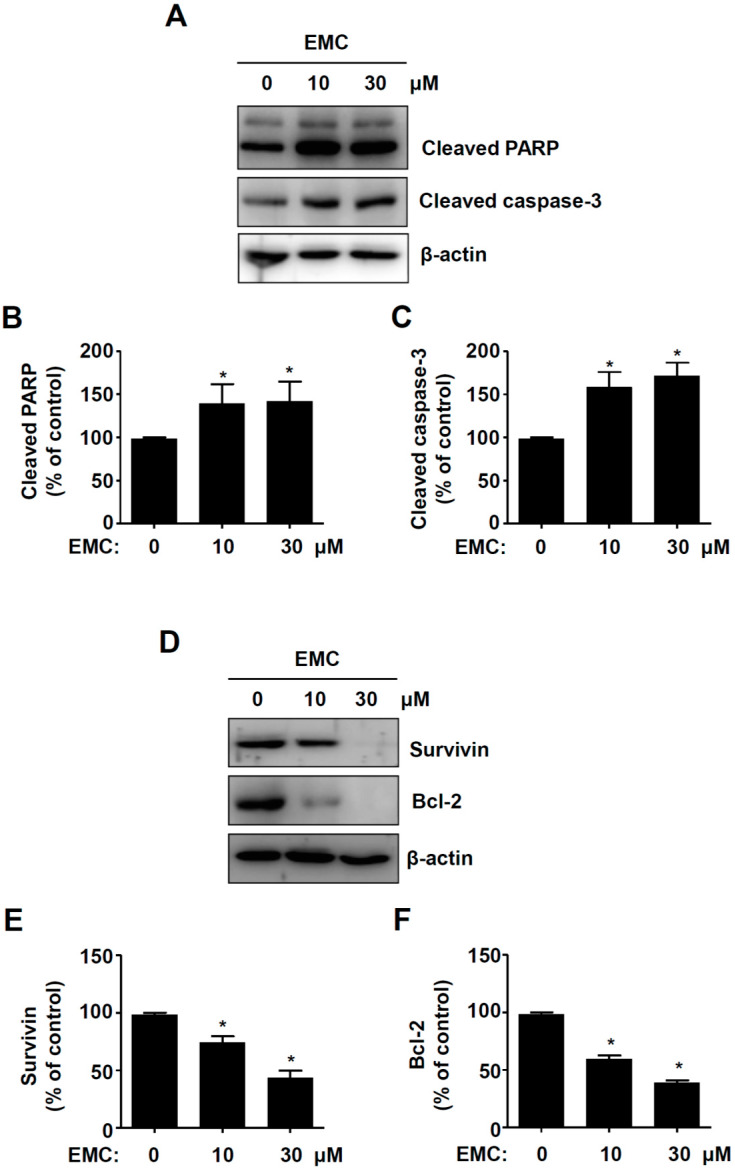
EMC induces apoptotic cell death in pre-osteoblasts. (**A**–**C**) Pre-osteoblasts were treated in the absence and presence of EMC for 24 h. They were analyzed by western blot analysis with antibodies against the cleaved PARP and caspase-3 (**A**). β-actin was detected on the same sample to normalize the number of lysates. The levels of the cleaved PARP (**B**) and caspase-3 (**C**) were represented as relative percentages of the control. (**D**–**F**) The cells were analyzed by western blot analysis using Survivin and Bcl-2 antibodies (**D**). After normalization by β-actin, the levels of Survivin (**E**) and Bcl-2 (**F**) were represented as relative percentages of the control. The data are expressed as the mean ± S.E.M. of experiments. * (*p* < 0.05) indicates statistically significant differences compared to the control.

**Figure 4:**
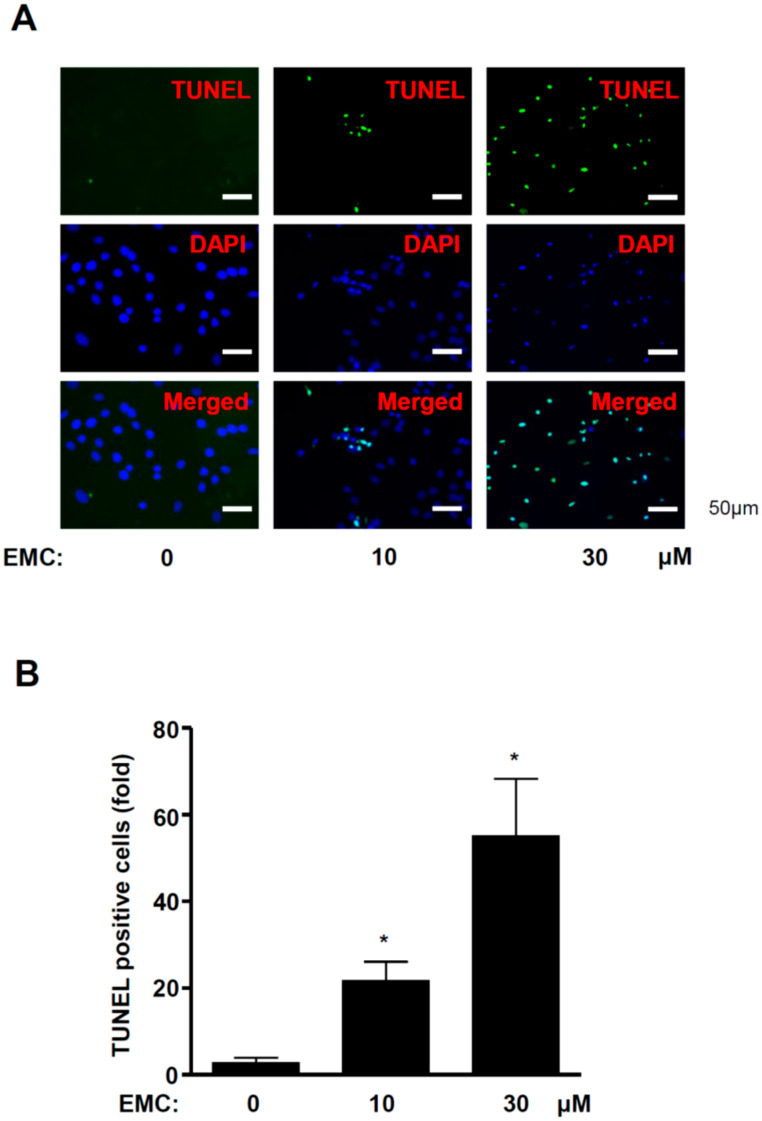
EMC increases DNA fragmentation in pre-osteoblasts. (**A**) DNA fragmentation was stained by TUNEL reaction (green) and DAPI (blue) solution at 24 h after EMC in pre-osteoblasts and observed by a confocal microscope. (B) The total number of cells in a given area was determined by DAPI staining. For quantification, three randomly selected areas were assessed. Scale bar: 50 µm. The results are representative of three independent experiments. The data are expressed as the mean ± S.E.M. of experiments. * (*p* < 0.05) indicates statistically significant differences compared to the control.

To determine whether the induction of apoptosis caused the inhibitory effects of EMC in pre-osteoblasts, we investigated the expression of apoptotic and anti-apoptotic proteins. EMC significantly increased the cleaved forms of poly (ADP-ribose) polymerase (PARP) and caspase-3 (Figure 3A–C), while it decreased the expression levels of Survivin and Bcl-2 (Figure 3D–F). We also demonstrated the process of apoptosis in pre-osteoblasts by EMC using the terminal deoxynucleotidyl transferase-mediated FITC–dUDP nick-end labeling (TUNEL) assay to detect DNA strand breaks and fragmentation. TUNEL-positive and DAPI-stained cells significantly increased in the presence of EMC in a dose-dependent manner (Figure 4A,B).

### 2.3. EMC Decreases MAPKs Signaling and Cell Migration in Pre-Osteoblasts

**Figure 5:**
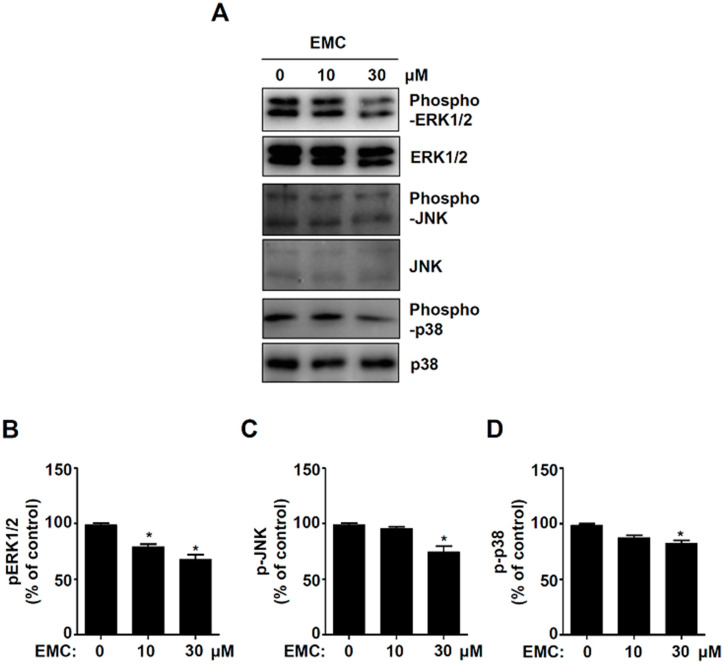
EMC decreases MAPKs signaling in pre-osteoblasts. (**A**–**D**) 24 h after EMC treatment, phoshpho-ERK1/2, ERK1/2, phospho-JNK, JNK, phospho-p38, and p38 were detected by western blot analysis. The levels of the phoshpho-ERK1/2 (**B**), phospho-JNK (**C**), and phospho-p38 (**D**) were represented as relative percentages of the control after normalization of the phosphorylation level by the total level (ERK1/2, JNK, and p38). The data are expressed as the mean ± S.E.M. of experiments. * (*p* < 0.05) indicates statistically significant differences compared to the control.

**Figure 6:**
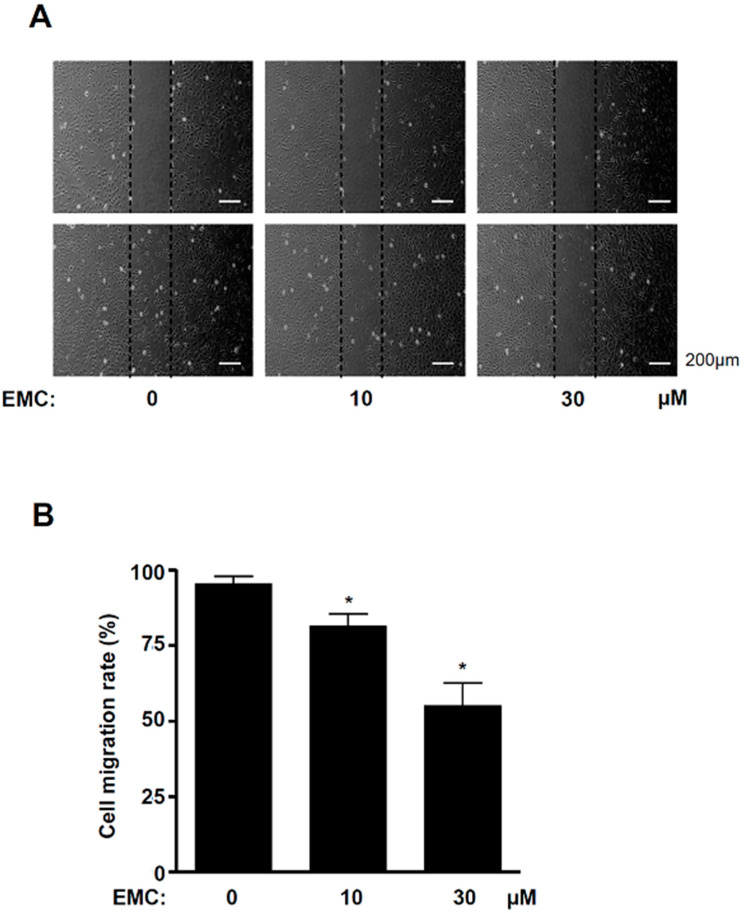
EMC reduces cell migration rate in pre-osteoblasts. (**A**,**B**) Pre-osteoblasts were treated with EMC for 24 h. Then, cell migration was observed under the phase contrast microscopy (**A**), and cell migration rate (%) was measured by tracing the area enclosing the spreading cell population. It was expressed as a bar graph normalized to the control. Scale bar: 200 µm. The results are representative of three independent experiments. The data are expressed as the mean ± S.E.M. of the experiments. * (*p* < 0.05) indicates statistically significant differences compared to the control.

Since the mitogen-activated protein kinases (MAPKs), including the ERK1/2, JNK, and p38 cascade prevent apoptosis, we examined the effects of EMC on the activity of MAPKs in pre-osteoblasts using western blot analysis. The results showed that EMC significantly inhibited the phosphorylation of ERK1/2 in a dose-dependent manner (Figure 5A.B). However, a significant phosphorylation reduction of JNK and p38 was exhibited only with 30 mM EMC (Figure 5A,C,D). To investigate whether EMC affects the migration of pre-osteoblasts due to apoptosis, we performed a wound-healing migration assay. EMC significantly suppressed the cell migration rate in a dose-dependent manner (Figure 6A,B).

### 2.4. EMC Suppresses the Osteoblast Differentiation of Pre-Osteoblasts

**Figure 7:**
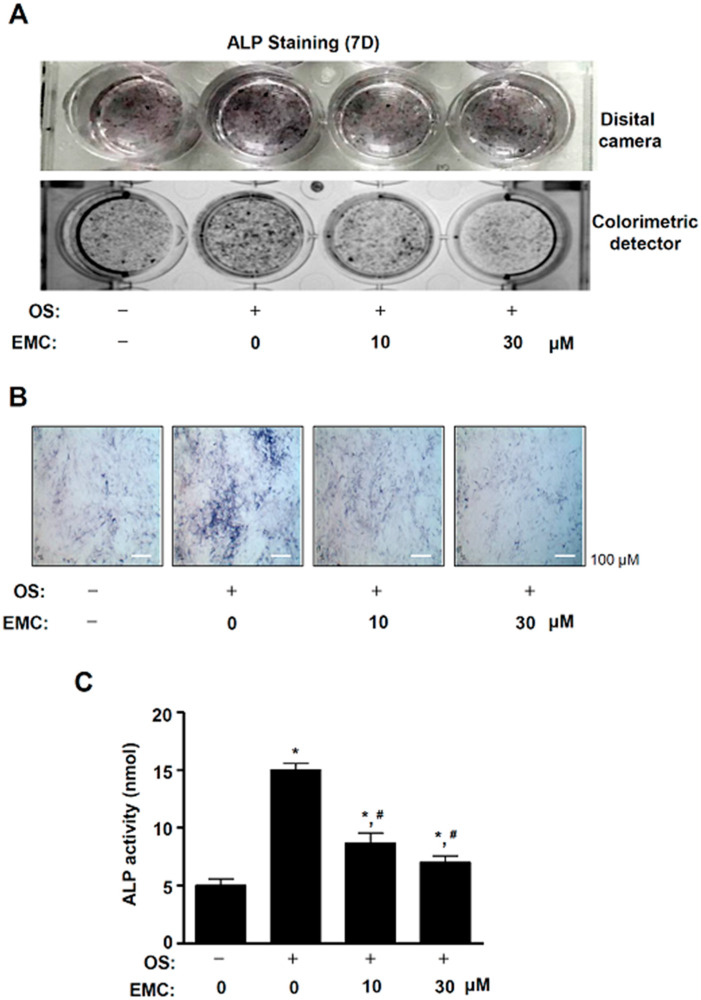
EMC suppresses the osteoblast differentiation of pre-osteoblasts. (**A**,**B**) Pre-osteoblasts were cultured in OS containing 50 µg/mL L-AA and 10 mM β-GP in the absence and presence of EMC for 7 days. Then ALP staining was observed by a disital camera (*upper*) and colorimetric detector (*bottom*) (**A**). The individual ALP-stained cells were detected using a light microscope (**B**). (**C**) Under the same condition, ALP activity was measured at 405 nm by the Multiskan GO Microplate Spectrophotometer. Scale bar: 100 µm. The results are representative of three independent experiments. The data are expressed as the mean ± S.E.M. of experiments. * (*p* < 0.05) indicates statistically significant differences compared to the control. # (*p* < 0.05) indicates statistically significant differences compared to the OS.

To examine the effects of EMC on the osteoblast differentiation of pre-osteoblasts, we induced osteoblast differentiation using an osteogenic supplement medium (OS) containing 50 µg/mL L-ascorbic acid (L-AA) and 10 mM β-glycerophosphate (β-GP) with EMC. The osteoblast differentiation was observed using Alkaline phosphatase (ALP) staining assays, a digital camera, and a colorimetric detector. EMC decreased the osteoblast differentiation induced by OS containing L-AA and β-GP (Figure 7A). The inhibition of osteoblast differentiation by EMC was also observed using a light microscope (Figure 7B). To validate these effects of EMC, we performed an ALP activity assay. EMC significantly decreased ALP activity under osteoblast differentiation in a dose-dependent manner (Figure 7C). EMC also suppressed the mRNA level of early and late osteogenic markers, ALP, osteocalcin (OCN), and osteopontin (OPN) (Supplementary Figure S1A–C).

## 3. Discussion

Bone pathophysiology is regulated by the proliferation, apoptosis, migration, and differentiation of osteoblasts [11,13,17]. The impairment of osteoblast function causes several bone diseases, such as osteoporosis, periodontal disease, osteopetrosis, and Paget’s disease [14,19,20,21]. Apoptosis is initially induced by the activation of caspase-8 and -9 through the extrinsic death receptor pathway and the intrinsic mitochondrial pathway, leading to the activation of executioner caspase-3 [22]. The activation of caspase-3 leads to the cleavage of the DNA repair enzyme PARP that is critical in the apoptotic process [23]. In the present study, we found that EMC activates caspase-3 and induces the cleavage of PARP in pre-osteoblasts. Activated caspase-3 also causes morphological changes, including cell shrinkage and chromatin condensation, and is governed by the Bcl-2 family and Survivin, which are considered anti-apoptotic components [22]. We also demonstrated that EMC downregulates Survivin and Bcl-2. It also induces apoptotic morphologic changes and DNA fragmentation in pre-osteoblasts. It was reported that caspase activity plays a crucial role in osteoblast apoptosis and survival, which affect bone development and metabolism. [24]. Our results suggest a critical role of EMC in regulating programmed cell death through apoptosis-related proteins in pre-osteoblasts.

Apoptotic cells lose the cell-to-cell adhesions, have reduced migration, and become separated from neighboring cells [22]. Cell migration and osteoblast differentiation also play an important role in bone metabolism and bone diseases [25]. The migration and differentiation of osteoblasts are required to form the appropriate shape of bones from the bone marrow, periosteum, surrounding tissues, and circulating blood [26]. In the present study, we demonstrated that EMC suppresses the migration rate of pre-osteoblasts and also inhibits the activation of ERK1/2, JNK, and p38. It was reported that the signaling of ERK1/2, JNK, and p38 controls cell proliferation, apoptosis, and migration. [27,28,29,30]. The migration and differentiation of osteoblast into bone tissue influences the synthesis, secretion, and calcium phosphate mineralization of the bone matrix [16,17,18]. We also demonstrated that EMC reduced ALP staining and activity during osteoblast differentiation. ALP is used as a marker of osteoblast differentiation since ALP is expressed early in bone development and is a key factor in the process of osteogenesis [31,32,33]. It was also reported that ALP expression is upregulated by the Smad1/5/8 and RUNX2 signaling pathways during osteogenesis [34,35]. Thus, our results suggest that EMC inhibits cell migration and osteoblast differentiation through the inactivation of ERK1/2, JNK, and p38, and the downregulation of ALP in pre-osteoblasts.

In conclusion, we originally reported evidence for the pharmacological effects and mechanisms of EMC isolated from the seeds of *A. katsumadai* in pre-osteoblasts. Our present results demonstrate that EMC increases cell apoptosis and decreases cell migration and osteoblast differentiation through the regulation of apoptotic signaling, and MAPKs and ALP activities in pre-osteoblasts. Our data also suggest that EMC might be a useful phytotherapeutic compound that could be used to treat abnormalities of osteoblast function in bone diseases.

## 4. Materials and Methods

### 4.1. Plant Material

The seeds of *A. katsumadai* were purchased from a commercial herbal market, Dongwoodang in Yeongcheon, Gyeongbuk, Korea, in March 2013. A voucher specimen (C537) has been deposited in the Natural Products Bank, National Institute for Korean Medicine Development (NIKOM).

### 4.2. Extraction and Isolation of Methyl-Cinnamate

The dried seeds of *A. katsumadai* (10.0 kg) were extracted exhaustively with 80% ethanol (EtOH:H_2_O = 9:1, 24 h × 2) at room temperature to yield the extract (1.0 kg). The crude extract was partitioned with water and organic solvents (*n*-hexane, ethyl acetate, and *n*-butanol). Ethyl acetate (EtOAc)-soluble extract (300.0 g of 399.9 g) was loaded on silica gel (70–230 mesh) open column chromatography with *n*-hexane/EtOAc (100:0 → 1:99, *v*/*v*; 500 mL each) to afford 10 subfractions (AKE 1~AKE 10). Subfraction AKE 2 (670.0 mg) was separated by silica gel preparative HPLC and eluted with a gradient system of *n*-hexane/EtOAc (9:1 → 2:8, *v*/*v*) to afford 10 subfractions (AKE 2–1~AKE 2–10) that were combined based on analytical HPLC analyses. Subfraction AKE 2–3 (133.0 mg) was separated using a Sephadex LH-20 column chromatography using methanol to yield six subfractions (AKE 2-3-1~AKE 2-3-6). The active compound (40.0 mg) was obtained from subfraction AKE 2-3-3 with gradient solvents of MeOH/H_2_O (50:50 → 20:80, *v*/*v*). The structure of (E)-methyl-cinnamate (EMC) was elucidated by comparing the spectral data based on previous literature [3].

### 4.3. (E)-Methyl-Cinnamate (EMC)

Colorless amorphous powder; electron ionization mass spectrometer (EI-MS) *m/z* 162 [M]^+^; Molecular formula C_10_H_10_O_2_; ^1^H-NMR (500 MHz, CD_3_OD) *δ* 7.67 (1H, d, *J* = 16.1 Hz, H-7), 7.57 (2H, m, H-2, H-6), 7.38 (3H, m, H-3, H-4, H-5), 6.50 (1H, d, *J* = 16.0 Hz, H-8), 3.77 (3H, s, OCH_3_); ^13^C-NMR (500 MHz, CD_3_OD) *δ* 169.1 (C-9), 146.4 (C-7), 135.8 (C-1), 131.6 (C-4), 130.1 (C-2, C-6), 129.4 (C-3, C-5), 118.7 (C-8), 52.3 (OCH_3_).

### 4.4. Nuclear Magnetic Resonance (NMR)

Nuclear magnetic resonance (NMR) experiments were performed on a JEOL ECX-500 spectrometer (^1^H, 500 MHz; ^13^C, 125 MHz; JEOL Ltd., Japan). All chemical shifts were referenced relative to the corresponding signals (δ_H_ 3.31/δ_C_ 49.15 for CD_3_OD). EI-MS data were obtained using micromass spectrum (AUTOSPEC, UK). High-performance liquid chromatography (HPLC) was performed using the Agilent 1200 series (Agilent Technologies, CA, USA). Open column chromatography (CC) was carried out over a silica gel 60 (70–230 mesh, 230–400 mesh ASTM, Merck, Darmstadt, Germany) and sephadex LH-20 gel (GE Healthcare, Sweden). Pre-coated silica gel 60 F_254_ (Merck) were used for thin-layer chromatography (TLC).

### 4.5. Culture of Pre-Osteoblast MC3T3-E1 Cells and Osteoblast Differentiation

Pre-osteoblast MC3T3E-1 cells (#CRL-2593) purchased from the American Type Culture Collection (ATCC) (Manassas, VA) were provided by the Bioevaluation Center (Korea Research Institute of Bioscience and Biotechnology, Republic of Korea). The cells were cultured in *α*-minimum essential medium (*α*-MEM) without L-ascorbic acid (WELGEME, Inc., Republic of Korea) supplemented with 10% fetal bovine serum (FBS), penicillin (100 units/mL), and streptomycin (100 µg/mL) at 37 °C in a humidified atmosphere of 5% CO_2_ and 95% air. The differentiation of osteoblast was induced by changing the osteogenic supplement medium (OS) containing 50 µg/mL L-ascorbic acid and 10 mM β-glycerophosphate (Sigma-Aldrich, St. Louis, MO). The medium was replaced every 2 days during the incubation period.

### 4.6. MTT Assay

Cell viability was measured using an MTT assay to detect NADH-dependent dehydrogenase activity as previously described in Reference [36].

### 4.7. Western Blot Analysis

Western blot analysis was carried out as previously described in Reference [37]. Briefly, equal amounts of proteins (20 µg) transferred to a polyvinylidene fluoride (PVDF) membrane (Millipore, Bedford, MA) were blocked for 1 hr at room temperature and incubated overnight at 4 °C with the specific primary antibodies. The membrane incubated with diluted horseradish peroxidase (HRP)-conjugated secondary antibodies (1:10,000, Jackson ImmunoResearch, West Grove, PA) for 2 h at room temperature was detected using the ProteinSimple detection system (ProteinSimple Inc., Santa Clara, CA).

### 4.8. TUNEL Assay

DNA fragmentation was examined using TUNEL. TUNEL assays were performed using the in situ Cell Death Detection Kit (Roche Diagnostics GmbH, Mannheim, Germany) according to the manufacturer’s instructions [38]. For 4′,6-diamidino-2-phenylindole (DAPI) (Sigma-Aldrich) staining, cells were incubated for 15 min at room temperature in the dark. TUNEL-positive and DAPI-stained cells were observed using a confocal microscope (K1-Fluo Confocal Laser Scanning Microscope, Republic of Korea).

### 4.9. Cell Migration Assay

Cell migration was assessed using an in vitro wound-healing assay [39]. The cells wounded with a 200 µL pipette tip were incubated in the absence and presence of EMC for 24 h at 37 °C in a humidified atmosphere of 5% CO_2_ and 95% air. Cell migration was observed using a light microscope, and the cell migration rate was quantified.

### 4.10. ALP Staining Assay

Cells were washed with 1 × PBS and then fixed in 10% formalin for 15 min at room temperature. After washing with distilled water, the cells were incubated with substrate solution for the reaction of ALP at 37 °C for 1 hr, according to the manufacturer’s protocol (Takara Bio Inc., Japan). The ALP staining was detected using a digital camera and colorimetric detector (ProteinSimple Inc., Santa Clara, CA).

### 4.11. ALP Activity Assay

The cell lysates were obtained using an alkaline phosphatase activity colorimetric assay kit (Biovision, Milpitas, CA) [36]. The absorbance was measured at 405 nm using the Multiskan GO Microplate Spectrophotometer (Thermo Fisher Scientific, Waltham, MA).

### 4.12. Real-Time PCR Analysis

Total RNA was extracted using the RNAqueous^®^ kit and cDNA synthesized from 1 µg of total RNA using the High Capacity RNA-to-cDNA kit (Applied Biosystems, Foster City, CA, USA) according to the manufacturer’s protocol. Primers were as follows: ALP forward: 5′-ACACCTTGACTGTGGTTACT-3′, ALP reverse: 5′-CCATATAGGATGGCCGTGAA-3′, OCN forward: 5′-GAGGTGATAGCTTGGCTTAT-3′, OCN reverse: 5′-TCCTTAGACTCACCGCTCTT-3′, OPN forward: 5′-ACACCATGAGGACCATCTTT-3′, OPN reverse: 5′-CGGAGTCTGTTCACTACCTT-3′, β-actin forward: 5′-AATGTGGCTGAGGACTTTGT-3′, β-actin reverse: 5′-GGGACTTCCTGTAACCACTT-3′. Quantitative real-time PCR was performed using a 7500 Real-Time PCR System (Applied Biosystems).

### 4.13. Statistical Analysis

The data were analyzed using the Prism Version 5 program (GraphPad Software, Inc., San Diego, CA). All numeric values are presented as the means ± S.E.M. The statistical significance of the data was determined using a Student’s unpaired *t*-test. A value of *p* < 0.05 was considered to indicate statistical significance.

## Supplementary Materials

Supplementary materials can be found at https://www.mdpi.com/1422-0067/21/10/3700/s1. Figure S1. Effects of EMC on Osteoblast differentiation. (A–C) Pre-osteoblasts were differentiated with the indicated concentrations of EMC for 7 days, and total RNA was isolated. Osteoblast-specific early and late marker genes including ALP (A), OCN (B), and OPN (C) were analyzed by qRT-PCR. The values obtained for the target gene expression were normalized to β-actin. The data are expressed as the mean ± S.E.M. of experiments. * (*p* < 0.05) indicates statistically significant differences compared to the control. # (*p* < 0.05) indicates statistically significant differences compared to the OS.

## Abbreviations

ALP	Alkaline phosphatase
β-GP	β -glycerophosphate
EMC	(E)-methyl-cinnamate
L-AA	L-ascorbic acid
MAPKs	Mitogen-activated protein kinases
MSCs	Mesenchymal stem cells
MTT	3-[4,5-dimethylthiazol-2-yl]-2,5-diphenyltetrazolium bromide (MTT)
OS	Osteogenic supplement
PARP	Poly (ADP-ribose) polymerase
RUNX2	Runt-related transcription factor 2
