# Supplementary material for: A Phytochemical Constituent, (E)-Methyl-Cinnamate Isolated from Alpinia katsumadai Hayata Suppresses Cell Survival, Migration, and Differentiation in Pre-Osteoblasts
Source: Int J Mol Sci. 2020 May 24;21(10):3700. doi: 10.3390/ijms21103700 (PMC7279157; doi:10.3390/ijms21103700)
Supplement: Supplementary file 1 [file ijms-21-03700-s001.pdf]

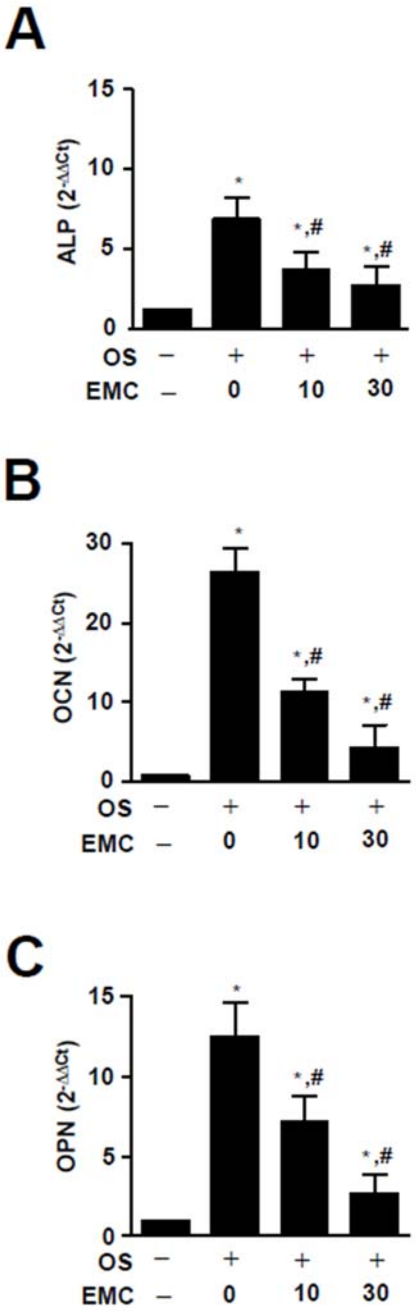

Supplementary Figure S1. Effects of EMC on Osteoblast differentiation. (A–C) Pre-osteoblasts were differentiated with the indicated concentrations of EMC for 7 days, and total RNA was isolated. Osteoblast-specific early and late marker genes including ALP (**A**), OCN (**B**), and OPN (**C**) were analyzed by qRT-PCR. The values obtained for the target gene expression were normalized to β-actin. The data are expressed as the mean ± S.E.M. of experiments. *(p < 0.05) indicates statistically significant differences compared to the control. # (p < 0.05) indicates statistically significant differences compared to the OS.
